# Supplementary material for: Towards sustainable solutions: Effective waste classification framework via enhanced deep convolutional neural networks
Source: PLoS One. 2025 Jun 4;20(6):e0324294. doi: 10.1371/journal.pone.0324294 (PMC12136365; doi:10.1371/journal.pone.0324294)
Supplement: Attachment — Submitted filename: Response letter--PONE-D-24-20413.pdf [file pone.0324294.s001.pdf]

Manuscript ID: PONE-D-24-20413

Article Title: Towards Sustainable Solutions: Effective Waste Classification Framework via Enhanced Deep Convolutional Neural Networks

Authors: Md. Minhazul Islam, S. M. Mahedy Hasan, Md. Rakib Hossain, Md. Palash Uddin and Md Al Mamun.

To: PLOS ONE Editor-in-Chief

Re: Responses to reviewers.

Dear Editor-in-Chief,

We would like to express our heartfelt gratitude to the academic editor and the reviewer for their valuable time and insightful feedback on our manuscript. We have carefully considered all the comments provided by the academic editor and the reviewer and have made the necessary revisions to the manuscript. This document details how we have responded to the reviewers' concerns. For the ease of the editor and reviewer, all changes in the revised manuscript are highlighted in red.

Thanks for your consideration!

Kind Regards,

Dr. Md Palash Uddin (On behalf of all co-authors)

To the Academic Editor

Comment#1: When submitting your revision, we need you to address these additional requirements. Please ensure that your manuscript meets PLOS ONE's style requirements, including those for file naming. The PLOS ONE style templates can be found at https://journals.plos.org/plosone/s/file?id=wjVg/PLOSOne_formatting_sample_main_body.pdf and https://journals.plos.org/plosone/s/file?id=ba62/PLOSOne_formatting_sample_title_authors_affiliations.pdf.

Author response: Thanks for reminding us to follow the journal formatting requirements.

Author action: We have written the entire paper in Latex using PLOS ONE's suggested template collected from the cite: https://journals.plos.org/plosone/s/latex. Additionally, we have also rechecked the format after generation of the pdf. As a result, we can confirm that the requirement is correctly satisfied.

Comment#2: Please note that PLOS ONE has specific guidelines on code sharing for submissions in which author-generated code underpins the findings in the manuscript. In these cases, all author-generated code must be made available without restrictions upon publication of the work. Please review our guidelines at https://journals.plos.org/plosone/s/materials-and-software-sharing#loc-sharing-code and ensure that your code is shared in a way that follows best practice and facilitates reproducibility and reuse.

Author response: Thanks for the comment.

Author action: Regarding the availability of the code, the authors assert that, due to ethical considerations, the code cannot be made publicly available. However, readers requiring access for manuscript assessment purposes may request the code. To do so, a formal request should be sent to the corresponding author.

Comment#3: Thank you for stating the following in your Competing Interests section: "NO authors have competing interests." Please complete your Competing Interests on the online submission form to state any Competing Interests. If you have no competing interests, please state "The authors have declared that no competing interests exist.", as detailed online in our guide for authors at http://journals.plos.org/plosone/s/submit-now

Author response: Thanks for the comment.

Author action: We confirm that there are no competing interests related to this manuscript. As requested, we have stated in the Competing Interests section that "Authors have no conflict of interest to declare."

Additional Comments:

Comment#4: The following limitations needs to be addressed: Despite using four publicly available datasets, the study may still lack sufficient diversity to fully represent global waste variations, especially in regions with unique waste profiles.

Author response: We sincerely thank the editor for providing this valuable suggestion.

Author action: We have addressed this limitation in the revised manuscript by expanding the "Threats to Validity" subsection. Specifically, on page 24, lines 941 to 942, we acknowledge the potential lack of diversity in the selected datasets.

Comment#5: The quality of images in the datasets might vary, which could impact the model's performance, especially in real-world scenarios where image quality may be inconsistent.

Author response: Thanks for the comment.

Author action: This limitation has been addressed in the "Threats to Validity" subsection on page 24, lines 942 to 944 in the revised manuscript.

Comment#6: While the model was successfully deployed in a web-based system, its scalability to larger, more complex systems or across different geographies remains untested.

Author response: We appreciate the editor's insightful suggestion and thanks for bringing this to our attention.

Author action: We acknowledge this limitation and have addressed it as well in the "Threats to Validity" subsection on page 25, lines 965 to 966 in the revised version.

Comment#7: The model's performance in diverse environmental conditions (e.g., different lighting or weather conditions) was not explicitly tested, which could affect real-world accuracy.

Author response: Thank you for your feedback.

Author action: We acknowledge this and have addressed it in the "Threats to Validity" subsection on page 25, lines 966 to 968 in the revised manuscript, just after the previous limitation discussed.

Comment#8: The evaluation was conducted on publicly available datasets rather than real-time data from waste management facilities, potentially limiting the model's real-world applicability.

Author response: We sincerely thank the editor for providing this valuable suggestion.

Author action: We have carefully revised the manuscript to address this limitation. The discussion can be found in the "Threats to Validity" subsection on page 24, lines 945 to 947.

Comment#9: The DenseNet201 architecture and the parallel CNN branches might require significant computational resources, which could be a barrier for deployment in resource-constrained environments.

Author response: Thanks for the comment.

Author action: This limitation has been addressed in the "Threats to Validity" subsection on page 24, lines 951 to 953.

Comment#10: The integration of DenseNet201 with SE mechanisms and parallel CNNs adds complexity, which might make the model more difficult to interpret and deploy.

Author response: Thank you for raising this issue.

Author action: Upon review, this limitation has been addressed in the "Threats to Validity" subsection on page 24, lines 954 to 955 in the revised version.

Comment#11: The study does not address potential latency issues in real-time waste classification, which could be critical for practical applications.

Author response: We appreciate the feedback on this important aspect.

Author action: We have reviewed and addressed this limitation in the "Threats to Validity" subsection on page 25, lines 968 to 970 in the revised manuscript, where we discuss potential latency issues in real-time waste classification.

Comment#12: The study does not explore the potential for deploying the model on edge devices, which could be important for on-site waste management in remote or under-resourced areas.

Author response: We sincerely thank the editor for this valuable comment.

Author action: Upon review, we found that this limitation has already been addressed in the original manuscript. It can now be found in the "Threats to Validity" subsection on page 25, lines 979 to 982 in the revised version.

Comment#13: The model might overfit to the specific datasets used for training, reducing its generalization capability to unseen data.

Author response: Thank you for bringing up this important concern.

Author action: Regarding the overfitting concern, the model did not encounter this issue while training with the four datasets, as illustrated in Figures 4, 8, 12, and 16, which show the accuracy and loss curves for the DenseNet201-Infused Parallel SE-CNN across the datasets. Additionally, early stopping has been

incorporated into the model, which helps prevent overfitting by halting training if the model begins to overfit on any specific dataset.

Comment#14: If the datasets used are biased towards certain types of waste, the model may not perform well on underrepresented waste types.

Author response: Thank you for highlighting this issue.

Author action: This limitation has been addressed in the "Threats to Validity" subsection on page 24, lines 947 to 948 in the revised manuscript.

Comment#15: The study does not thoroughly investigate the model's robustness to adversarial attacks or noisy data, which are common in real-world scenarios.

Author response: Thank you for pointing this out.

Author action: We acknowledge this limitation and have addressed it in the "Threats to Validity" subsection on page 24, lines 955 to 956 in the revised manuscript.

Comment#16: The energy consumption of the model, especially in large-scale deployments, is not addressed, which could be a significant factor in sustainable waste management.

Author response: Thank you for highlighting this important aspect.

Author action: This limitation was not addressed previously. In the new version, it has been added to the "Threats to Validity" subsection on page 25, lines 968 to 970.

Comment#17: While the study introduces a novel approach, it may not include comprehensive comparisons with a wide range of existing waste classification methods.

Author response: Thanks for the comment.

Author action: The study includes a thorough comparison with a wide range of existing waste classification methods, as detailed in the original manuscript. In the revised version, this is presented in the "Performance Comparison with Different Deep CNN Architectures and State of the Art Researches" subsection under the "Results Analysis" section. Table 9 provides a comparative analysis of classification performance among models from prior studies for waste classification purposes, as discussed on page 21. Additionally, a detailed description is available on pages 21 to 22, lines 820 to 851.

Comment#18: The long-term stability and performance of the model over time, particularly as waste compositions change, have not been explored.

Author response: We sincerely thank the editor for providing this comment.

Author action: We acknowledge this limitation, and it has been addressed in the revised paper in the "Threats to Validity" subsection on page 25, lines 970 to 971.

Comment#19: The cost-effectiveness of implementing this advanced model in various waste management contexts has not been analyzed.

Author response: Thank you for raising this important issue.

Author action: This issue has been addressed in the revised manuscript in the "Threats to Validity" subsection on page 25, lines 974 to 975.

Comment#20: The complexity of the model may require specialized training for users, which could be a barrier to widespread adoption.

Author response: Thanks for the comment.

Author action: This limitation is acknowledged in the revised manuscript in the "Threats to Validity" subsection on page 25, lines 976 to 977.

Comment#21: The model may not be easily adaptable to specific local waste management needs, which could limit its effectiveness in diverse settings.

Author response: Thank you for highlighting this concern.

Author action: This limitation has been addressed as well after the previous limitation in the revised manuscript and can be found in the "Threats to Validity" subsection on page 25, lines 977 to 978.

Comment#22: The deployment of AI in waste management could raise ethical and privacy concerns, especially if data from waste is linked to individuals or households.

Author response: Thanks for the comment.

Author action: This concern has been addressed in the revised manuscript and can be found in the "Threats to Validity" subsection on page 25, lines 982 to 983.

Comment#23: The web-based deployment of the model assumes reliable internet access, which may not be available in all areas, particularly in remote or developing regions.

Author response: Thank you for pointing this out.

Author action: This issue has also been addressed in the revised manuscript, following the discussion of previous limitations, and can be found in the "Threats to Validity" subsection on page 25, lines 983 to 984.

To Reviewer 1

Reviewer#1, Comment#1: The abstract needs modification.

Author response: We appreciate the reviewer's feedback regarding the need to modify the abstract.

Author action: Although the reviewer did not specify the exact modifications required, we have proactively made some changes to enhance the abstract based on our understanding. These modifications can be found in the revised manuscript in the abstract section, specifically on page 1, lines 2 to 10 and 13 to 16.

Reviewer#1, Comment#2: The references are to be cited properly

Author response: We greatly appreciate the reviewer's comment regarding the citation of references.

Author action: The references have been carefully reviewed, and all citation issues have been corrected. Specifically, there was an error where the author's first name was used instead of the last name in a sentence, and one reference was incorrectly placed in an unrelated sentence. The corrections can be found on page 5, line 210, and on page 2, lines 45 to 46, respectively. Additionally, the contributor names were missing in three dataset references, and this has been corrected. The changes are located on page 28, from lines 1106 to 1111.

Reviewer#1, Comment#3: The research gap and limitations are to be included.

Author response: We sincerely appreciate the reviewer's comment.

Author action: The research gap and limitations regarding waste classification works can be found in the "Related Work" section of the manuscript, spanning from page 3 to page 5, lines 121 to 228. Various previous works and their limitations are discussed throughout this section. To further clarify, a concise paragraph has been added on page 5 to 6, lines 229 to 241, in the revised manuscript to provide an overview summary of the research gaps and limitations in previous studies that we aimed to address. As for the limitations of our study, these can be found in the "Threats to Validity" section on pages 24 to 25, lines 937 to 984.

Reviewer#1, Comment#4: A tree diagram may be added.

Author response: We sincerely appreciate the reviewer's valuable suggestion on adding a tree diagram.

Author action: We have already included a detailed diagram of the architecture in the manuscript, specifically in Figure 2, which we believe effectively illustrates the model's structure and functionality. Adding a tree diagram might lead to redundancy without adding significant value to the explanation. Therefore, we have chosen not to include an additional tree diagram. We hope that this addresses your concern satisfactorily.

Reviewer#1, Comment#5: The figure title and legends are missing.

Author response: We sincerely appreciate the reviewer's comment.

Author action: We thoroughly checked to determine if any figure titles or legends were missing. Upon investigation, we found that no figure titles were missing. However, some figures were missing legends, specifically those showing the overall metrics overview for the 4 datasets. The necessary changes have been made in Figures 6, 10, 14, 18 to include the legends in the revised manuscript.

Reviewer#1, Comment#6: MCC, Kappa and GDR may be included.

Author response: We sincerely appreciate the reviewer for suggesting the inclusion of MCC, Kappa, and GDR in our study.

Author action: As per the suggestion, we have included MCC, Kappa, and GDR for the 4 datasets to ensure a rigorous analysis. The values are shown in the updated figures for the overall metrics overview in Figures 6, 10, 14, and 18 for the 4 datasets. The necessary equations for MCC, Kappa, and GDR have been provided in the "Performance Evaluation Measures" subsection under the "Research Methodology" section on page 10, from lines 415 to 433 in the revised manuscript.

For the "Waste Classification V2" dataset, the description regarding these metrics has been added in the "Performance Analysis of Waste Classification V2 Dataset" subsection under the "Result Analysis" section from lines 523 to 525 and 532 to 536 on page 13. For the "Waste Classification" dataset, the description has been added in the "Performance Analysis of Waste Classification Dataset" subsection under the "Result Analysis" section from lines 613 to 617 on page 15-16. For the "OpenRecycle" dataset, the description is included in the "Performance Analysis of OpenRecycle Dataset" subsection under the "Result Analysis" section from lines 690 to 694 on page 17-18. For the "TrashNet" dataset, the description has been added in the "Performance Analysis of TrashNet Dataset" subsection under the "Result Analysis" section from lines 753 to 756 on page 19 in the revised manuscript.

Reviewer#1, Comment#7: Time complexity may be analyzed.

Author response: We thank the reviewer for his suggestion to consider the analysis of time complexity in our study.

Author action: As per the valuable suggestion, we have added a "Brief Overview of Empirical Time Complexity" subsection in the "Result Analysis" section to ensure that rigorous analysis has been conducted. A table, titled "Empirical Time Complexity Analysis Across Datasets," has been included as Table 10. This table provides a brief empirical overview to give an overall idea of the model's time complexity across the datasets. The section spans pages 22 and 23, lines 852 to 876 in the revised manuscript, with a detailed description regarding this analysis.

Reviewer#1, Comment#8: A comparison with ML classifier will be included.

Author response: We appreciate the reviewer's comment to include a comparison with ML classifiers in our study.

Author action: As per the valuable comment, a comparison with ML classifiers has been included in the "Performance Comparison with Different Deep CNN Architectures and State-of-the-Art Research" subsection in the "Result Analysis" section. A table, titled "ML Classifiers Performance Comparison Across Datasets," has been added as Table 8. This table shows the results of classification performed with ML classifiers after feature extraction through the custom architecture, rather than using the proposed method with fully connected layers. A detailed analysis is provided on pages 20 and 21, from lines 803 to 819 in the revised manuscript.
