# Supplementary material for: Towards sustainable solutions: Effective waste classification framework via enhanced deep convolutional neural networks
Source: PLoS One. 2025 Jun 4;20(6):e0324294. doi: 10.1371/journal.pone.0324294 (PMC12136365; doi:10.1371/journal.pone.0324294)
Supplement: Attachment — Submitted filename: Response letter--PONE-D-24-20413R1.pdf [file pone.0324294.s002.pdf]

Manuscript ID: PONE-D-24-20413

Article Title: Towards Sustainable Solutions: Effective Waste Classification Framework via Enhanced Deep Convolutional Neural Networks

Authors: Md. Minhazul Islam, S. M. Mahedy Hasan, Md. Rakib Hossain, Md. Palash Uddin and Md Al Mamun.

To: PLOS ONE Editor-in-Chief

Re: Responses to reviewers.

Dear Editor-in-Chief,

We sincerely thank the academic editor for their valuable time and insightful feedback on our manuscript. We have carefully considered all the comments provided, recognizing that the previous version did not fully meet expectations. We have made the necessary revisions to address these concerns. This document outlines how we have responded to the editor's suggestions. For the ease of the editor, all changes in the revised manuscript are highlighted in red.

Thanks for your consideration!

Kind Regards,

Dr. Md Palash Uddin (On behalf of all co-authors)

To the Academic Editor

Comment#1: The following limitations needs to be addressed: Despite using four publicly available datasets, the study may still lack sufficient diversity to fully represent global waste variations, especially in regions with unique waste profiles.

Author response: We sincerely thank the editor for providing this valuable suggestion.

Author action: We appreciate the editor's observation regarding dataset diversity and acknowledge the potential limitations in fully representing global waste variations. To address this, we have expanded our evaluation by incorporating three additional publicly available datasets. These were tested under the same conditions as our primary datasets to assess the model's performance on a broader range of waste types. With the inclusion of these three datasets, we now have a total of seven datasets, covering 27 unique waste classes. This enhancement is detailed in the revised manuscript in the "Dataset" section (page 6, lines 255–269), and Table 2 on page 7 highlights the unique waste classes across these datasets.

Furthermore, the results from testing these additional datasets are provided in the "Validation Across Additional Datasets" subsection of the “Results Analysis” section (pages 20–21, lines 779–793), along with a performance metrics table showcasing the model’s effectiveness across these diverse waste classes. As mentioned in the manuscript, most previous studies rely on a single dataset; therefore, our work addresses this limitation by incorporating more diverse datasets to better showcase the model’s potential, which has been our goal from the beginning.

While we recognize that this may still not encompass all global waste variations, we believe that the inclusion of 27 distinct classes provides significant coverage of common waste categories. Additionally, as noted in the initial original manuscript, we have acknowledged in the "Threats to Validity" subsection (page 21, lines 840–842) that future work could further enhance dataset diversity by collecting more field-specific data from varied regions.

Comment#2: The quality of images in the datasets might vary, which could impact the model’s performance, especially in real-world scenarios where image quality may be inconsistent.

Author response: Thanks for the comment.

Author action: We appreciate the editor’s insightful comment on the potential impact of image quality variation across datasets. To address this concern and better simulate real-world conditions with inconsistent image quality, we trained the model on raw, unprocessed images across all seven datasets, adjusting only the image size without applying any enhancement techniques. In the initial manuscript, the first paragraph of the "Research Methodology" section on page 6 indicates that no other modifications were made to the data beyond resizing. In the revised manuscript, we further clarified this approach in the first paragraph of the same section on page 7, highlighting our intent to evaluate the model's performance under realistic conditions using raw, unprocessed images. Our rationale was that if the model can achieve high accuracy with raw, unenhanced images, it should perform even more robustly with enhanced datasets or refined inputs. By using raw images, we aimed to better prepare the model for real-world scenarios with varying image quality.

Comment#3: While the model was successfully deployed in a web-based system, its scalability to larger, more complex systems or across different geographies remains untested.

Author response: We appreciate the editor's insightful suggestion and thanks for bringing this to our attention.

Author action: We acknowledge this limitation, and in the initial manuscript, the "Introduction" section (page 3, lines 96–100) explicitly states that the web system is currently hosted on a local server, demonstrating its potential for broader deployment. Furthermore, in the "Threats to Validity" subsection in the initial manuscript (page 22, lines 849–853), we outline our future efforts to enhance the system's scalability to larger, more complex environments by extending deployment to reach a wider audience. We remain committed to addressing this limitation in our future work, as we believe that scaling the model will enhance its adaptability to varied environments and validate its effectiveness across a broader range of applications.

Comment#4: The model's performance in diverse environmental conditions (e.g., different lighting or weather conditions) was not explicitly tested, which could affect real-world accuracy.

Author response: Thank you for your feedback.

Author action: We appreciate your feedback regarding the model's performance under diverse environmental conditions. As previously mentioned, the model was initially tested using raw, unprocessed images to better simulate real-world situations. However, we acknowledge that rigorously evaluating the model's performance under varying lighting and weather conditions is crucial for accurately reflecting real-world scenarios.

To address this, we further evaluated the model by introducing variations in brightness, contrast, saturation, hue, gamma correction, and slight random sharpening within a moderate range. These adjustments more clearly simulate real-world variations. In the revised manuscript, we have included a detailed subsection titled "Robustness Evaluation under Adversarial Attacks and Environmental Variability" in the "Results Analysis" section on page 20. This subsection analyzes the model's performance following these variations. The results, including any accuracy drop due to these adjustments, are presented in Table 9 on page 20 of the revised manuscript. We hope this additional analysis effectively addresses your concerns.

Comment#5: The evaluation was conducted on publicly available datasets rather than real-time data from waste management facilities, potentially limiting the model's real-world applicability.

Author response: We sincerely thank the editor for highlighting the potential limitations regarding real-world applicability due to the use of publicly available datasets.

Author action: We acknowledge that testing data from real-world waste management facilities would provide valuable insights into the model's practical performance. In the original manuscript, we discussed the need for a more diverse and field-specific dataset in the "Threats to Validity" subsection (page 22, lines 840–842), recognizing this as a limitation of our study. However, we did not specifically highlight the importance of real-time data from waste management facilities, which we have now addressed in the revised manuscript in the "Threats to Validity" section on page 27, lines 1002–1003. This addition emphasizes the significance of evaluating model performance in diverse, real-world environments.

Although real-time data was unavailable for this study, we utilized raw images for testing the model. Additionally, we took further steps to approximate real-world conditions more accurately in the revised manuscript, specifically in the "Robustness Evaluation under Adversarial Attacks and Environmental Variability" subsection on page 20. In this subsection, we introduced environmental variations, such as adjustments in lighting and image quality, as previously mentioned. Moving forward, we plan to collaborate with waste management facilities to assess the model's performance using real-time data streams.

Comment#6: The DenseNet201 architecture and the parallel CNN branches might require significant computational resources, which could be a barrier for deployment in resource-constrained environments.

Author response: We appreciate your comment on the computational resource requirements associated with the DenseNet201 architecture and parallel CNN branches.

Author action: We acknowledge this issue as well. In the original manuscript, we discussed in the "Threats to Validity" subsection (page 22, lines 843–845) that the extended training period of models may pose challenges in resource-constrained settings, as it indicates that the model requires significant computational resources due to its complexity. In the revised manuscript, we have included the total number of parameters for the proposed model, along with additional deep CNN models that we evaluated, to assist in identifying optimal choices for deployment. This information can be found in Table 11, located in the "Performance Comparison with Different Deep CNN Architectures and State of the Art Researches" subsection of the "Results Analysis" section on page 22 of the revised manuscript.

From this table, it is evident that models with significantly fewer parameters do not perform as well as the proposed model. However, we have identified three alternative deep CNN models, which have approximately 1/3 to 1/2 the parameters of the proposed model. While these alternatives do not achieve the same level of accuracy as the proposed model, they still demonstrate good performance across all four datasets. These models can be suitable choices for resource-constrained environments, albeit with a trade-off in accuracy. This discussion can be found in the same subsection on page 21, lines 810–823 of the revised manuscript.

Comment#7: The integration of DenseNet201 with SE mechanisms and parallel CNNs adds complexity, which might make the model more difficult to interpret and deploy.

Author response: Thank you for raising this issue.

Author action: We acknowledge this concern and have addressed it in the "Threats to Validity" subsection of the original manuscript, noting that the model requires significant training time for larger datasets due to its complexity. This complexity does present challenges for interpretation and deployment. In the revised manuscript, we have further clarified this point, emphasizing that deployment can be difficult due to the model's complexity (page 27, lines 1007–1010).

Additionally, as mentioned previously, we identified three alternative deep CNN models in the discussion section on page 21 (lines 810–823) and presented them in Table 11 on page 22 of the revised manuscript. These alternative models have approximately 1/3 to 1/2 the parameters of the proposed model and are less complex. Two of these models, which do not incorporate parallel SE-CNN mechanisms, can be deployed more easily as they are less complex and easier to interpret, albeit at the cost of some accuracy compared to the proposed model. Moving forward, we aim to identify models with lower complexity that can achieve similar or even better accuracy than the proposed model.

Comment#8: The study does not address potential latency issues in real-time waste classification, which could be critical for practical applications.

Author response: We appreciate the feedback on this important aspect.

Author action: In the previous version of the revised manuscript, we introduced a "Brief Overview of Empirical Time Complexity" subsection within the “Results Analysis” section, where we provided a detailed analysis of the model’s time complexity across four datasets. This subsection includes the model's inference time, which measures the duration required to make predictions on new data. The analysis, along

with a comprehensive description, is available on page 24, lines 881–904 in the latest revised manuscript version, offering an estimate of potential latency based on the model's performance on the test set.

To better assess real-world latency, we also conducted an end-to-end latency evaluation by making predictions via the website in real time. This analysis includes factors such as image upload time and server processing time, offering a more accurate measure of real-time delay. As expected, the results indicate that end-to-end latency exceeds the model's standalone inference time. This evaluation, with further details, is now included in the "Website Deployment" subsection under the "Real-Time Deployment of Waste Classification System" section on page 26, lines 967–973 of the latest revised manuscript.. This addition offers a practical perspective on potential latency issues in real-time waste classification.

Comment#9: The study does not explore the potential for deploying the model on edge devices, which could be important for on-site waste management in remote or under-resourced areas.

Author response: We sincerely thank the editor for this valuable comment.

Author action: We acknowledge this limitation of our study, which we have already addressed in the original manuscript in the "Threats to Validity" subsection (page 22, lines 853–855). In the revised version, this information is now located in the “Threats to Validity” section on page 27 (lines 1018–1020). Moving forward, we aim to explore the potential for deploying our model on edge devices, as mentioned in the "Conclusion and Future Research " section as well.

Comment#10: The model might overfit to the specific datasets used for training, reducing its generalization capability to unseen data.

Author response: Thank you for bringing up this important concern.

Author action: Regarding the overfitting issue, our model did not encounter this problem while training on the four datasets, as illustrated in Figures 4, 8, 12, and 16, which display the accuracy and loss curves for the DenseNet201-Infused Parallel SE-CNN across the datasets. Additionally, we mentioned in the original manuscript that early stopping has been incorporated into the model. This technique helps prevent overfitting by halting training if the model begins to overfit on any specific dataset. This information can be found on page 10, lines 407–409, in the original manuscript, and it is located on page 11 in the revised manuscript.

Comment#11: If the datasets used are biased towards certain types of waste, the model may not perform well on underrepresented waste types.

Author response: Thank you for highlighting this issue.

Author action: We acknowledge that there is a possibility of bias in the datasets, particularly due to the lack of class balance. When certain classes have a significantly higher number of samples compared to others, the model can become biased towards those prevalent classes. We encountered this issue with the TrashNet dataset, where the model performed well across most classes but struggled with the "Trash" class due to its low number of images. This imbalance is a common challenge in real-world applications. Although the overall performance remained satisfactory, the poor representation of this class posed a problem.

To address this imbalance, we applied data augmentation techniques to increase the image count for the "Trash" class. This approach allowed the model to perform well across all classes without introducing bias toward specific waste types. In the revised manuscript, we provide a detailed analysis of this issue in the "Performance Analysis of TrashNet Dataset" subsection on page 19 (lines 710–731). This section includes a table showing class-wise metrics after data augmentation, clearly illustrating the improvements achieved through various augmentation techniques. While this method does add further complexity, it provides a feasible solution for datasets encountering similar challenges if required.

Comment#12: The study does not thoroughly investigate the model's robustness to adversarial attacks or noisy data, which are common in real-world scenarios.

Author response: Thank you for pointing this out.

Author action: We understand the importance of evaluating the model's robustness to adversarial attacks and noisy data, which we had not thoroughly investigated previously. While our initial evaluation used raw datasets in their original form, we recognize that a more rigorous assessment is essential to gauge the model's performance in challenging conditions. To address this, we have introduced a new subsection titled "Robustness Evaluation under Adversarial Attacks and Environmental Variability" in the "Results Analysis section" of the revised manuscript.

This subsection evaluates the model's performance under moderate levels of noise, such as salt-and-pepper noise, Gaussian noise, and FGSM adversarial attacks, to simulate real-world scenarios. The results, including relevant performance metrics and accuracy reductions in the presence of noise and adversarial inputs, are presented in Table 9 on page 20, with further discussion in the accompanying subsection. We hope this addresses the concern regarding the model's robustness to adversarial attacks and noisy data.

Comment#13: The energy consumption of the model, especially in large-scale deployments, is not addressed, which could be a significant factor in sustainable waste management.

Author response: Thank you for highlighting this important aspect.

Author action: We appreciate the reviewer's emphasis on the model's energy consumption, particularly regarding sustainable waste management. To address this, we evaluated the model's energy consumption and estimated carbon emissions across the five datasets. The findings are summarized in Table 15 on page 25 of the revised manuscript, with further details provided in the newly added "Energy Consumption Assessment" subsection within the "Results Analysis" section. This assessment serves as a preliminary indication of the model's energy efficiency and environmental impact, which are crucial for sustainable applications.

In the "Threats to Validity" section and the "Conclusion and Future Research" section, we have noted our intention to focus on broader deployment in future work, which will enable a more comprehensive assessment of the model's energy consumption at scale. Our evaluation of energy consumption, included in the revised manuscript, offers a valuable foundation for understanding the model's energy requirements within our current scope and their implications for environmentally responsible waste management systems. We hope this adequately addresses the editor's concern.

Comment#14: While the study introduces a novel approach, it may not include comprehensive comparisons with a wide range of existing waste classification methods.

Author response: Thanks for the comment.

Author action: We appreciate the opportunity to clarify that our study includes a comprehensive comparison of our model with a wide range of existing waste classification methods. In the original manuscript, this is presented in the "Performance Comparison with Different Deep CNN Architectures and State of the Art Researches" subsection within the “Results Analysis” section, specifically in Table 8 on page 19.

In the revised manuscript, Table 13 on page 23 presents a comparative analysis of classification performance across models from prior studies, including our proposed model evaluated on seven datasets. we have expanded our analysis to include additional comparisons. Additionally, a detailed description of these comparisons can be found on pages 22 to 24 (lines 847 to 880). Furthermore, we have evaluated the proposed model against other deep CNN architectures and machine learning classifiers, as shown in Tables 11 and 12 on page 22. This highlights that our study includes thorough comparisons with previous research and various models, enabling us to identify the optimal solution for waste classification.

Comment#15: The long-term stability and performance of the model over time, particularly as waste compositions change, have not been explored.

Author response: We sincerely thank the editor for providing this comment.

Author action: While we recognize the importance of long-term stability and performance of the model as waste compositions change, it is important to note that exploring this aspect was not within the primary scope of our study. Our main objective was to develop and evaluate an effective waste classification model using the specified datasets, covering global waste variations as much as possible. Additionally, we aimed to demonstrate potential, simpler deployment strategies to facilitate broader adoption. However, we acknowledge that understanding the model's performance over time could be a valuable area for future research. We appreciate this suggestion and will consider investigating the long-term stability and adaptability of the model in subsequent studies.

Comment#16: The cost-effectiveness of implementing this advanced model in various waste management contexts has not been analyzed.

Author response: Thank you for raising this important issue.

Author action: We appreciate the insight regarding the cost-effectiveness of implementing our advanced model in various waste management contexts. While a comprehensive economic analysis was beyond the initial scope of our study, our primary aim was to develop and rigorously evaluate the model's performance alongside effective deployment strategies. Importantly, the model was trained using Kaggle’s GPU, which is available at no cost, and the deployment methods we employed, such as Flask and Gradio, incur no additional financial investment. This accessibility may facilitate adoption by organizations with limited resources.

We acknowledge the significance of assessing the cost implications for implementing this technology in practice and will consider this aspect in our future research endeavors as we explore broader applications of our work. Thank you for highlighting this important consideration.

Comment#17: The complexity of the model may require specialized training for users, which could be a barrier to widespread adoption.

Author response: Thanks for the comment.

Author action: Thank you for highlighting this important consideration regarding user training. While the DenseNet201-infused SE-CNN architecture is indeed complex, we have implemented a web-based deployment using Flask to make user interaction straightforward. The web interface provides a seamless experience, where users simply upload images, and the system takes care of all processing steps. Furthermore, the entire workflow—from image input to prediction output—is fully automated, ensuring ease of use without requiring specialized knowledge. This design mitigates the need for user training, as it is intuitive and accessible.

In addition, we explored Gradio as an alternative deployment option to enhance accessibility further. Gradio, a Python-based tool, simplifies the deployment process by providing a high-level, user-friendly interface that automates the workflow entirely, eliminating the complexities of conventional web development. With both Flask and Gradio, users can leverage the model's capabilities effectively, supporting broader adoption without much need for any kind of user training. For a detailed overview of these deployment strategies, please refer to the section titled "Real-Time Deployment of Waste Classification System."

Comment#18: The model may not be easily adaptable to specific local waste management needs, which could limit its effectiveness in diverse settings.

Author response: Thank you for highlighting this concern.

Author action: We appreciate the reviewer's concerns regarding the adaptability of our model to diverse local waste management contexts. To clarify, the model has been rigorously tested on four datasets, with three additional datasets included in the revised manuscript, covering 27 distinct classes of waste as detailed in the "Dataset section." The model achieved accuracy rates ranging from 92.77% to 97.4%. Its performance on datasets of varying sizes, as presented in the "Result Analysis" section of the revised manuscript, demonstrates its scalability, maintaining high accuracy even with smaller, more complex datasets.

Given that many waste categories are universal, this broad class coverage ensures the model's effectiveness in most waste management contexts. However, we acknowledge that there may be local-specific waste types. In the future, we aim to collect diverse, field-specific datasets, as noted in the "Threats to Validity" subsection of the original manuscript (page 22, lines 840–842) and the "Conclusion and Future Research" section (lines 878–880). While gathering comprehensive regional data can be challenging, the existing universal waste categories provide a solid foundation for generalizing a model that can be effective on a global scale.

Comment#19: The deployment of AI in waste management could raise ethical and privacy concerns, especially if data from waste is linked to individuals or households.

Author response: Thanks for the comment.

Author action: The editor's concerns regarding the ethical and privacy implications of deploying AI for waste management, particularly the risk of linking waste data to individuals or households, are appreciated. However, it is important to clarify that this study exclusively utilizes public, anonymized datasets, with

references provided in the “Data Availability” part of the revised manuscript (lines 1055–1056). These datasets focus solely on the visual characteristics of waste, with no personal or household-level information involved in either the dataset or the classification process, ensuring complete privacy protection.

The model processes only waste images and does not collect or analyze any personally identifiable information (PII) at any stage of the research, including the deployment stage. During deployment, the trained deep learning model, saved in HDF5 format, is utilized to provide a prediction of the type of waste based solely on the uploaded image, as discussed in the “Website Deployment” subsection. The system is intended for general waste classification, contributing to broader waste management solutions without posing any privacy risks. The commitment to upholding these ethical standards will continue, especially as the model's capabilities and reach expand within waste management systems.

Comment#20: The web-based deployment of the model assumes reliable internet access, which may not be available in all areas, particularly in remote or developing regions.

Author response: Thank you for pointing this out.

Author action: The current deployment of the model using Flask enables it to run on a local machine without requiring internet access, providing the necessary flexibility for offline use. This means that users in areas with limited or unreliable connectivity can still utilize the system by running it locally. Although the Flask-based approach was discussed in the original manuscript under the "Real-Time Deployment of Waste Classification Systems" section on page 20, we did not specifically mention this offline functionality. To address this oversight, we have clarified this capability in the revised manuscript, located on page 25, lines 942–944, within the "Website Deployment" subsection. We hope this adequately addresses the editor's concern.
