# Supplementary material for: Towards sustainable solutions: Effective waste classification framework via enhanced deep convolutional neural networks
Source: PLoS One. 2025 Jun 4;20(6):e0324294. doi: 10.1371/journal.pone.0324294 (PMC12136365; doi:10.1371/journal.pone.0324294)
Supplement: Attachment — Submitted filename: Response letter--PONE-D-24-20413--2.pdf [file pone.0324294.s003.pdf]

Manuscript ID: PONE-D-24-20413

Article Title: Towards Sustainable Solutions: Effective Waste Classification Framework via Enhanced Deep Convolutional Neural Networks

Authors: Md Minhazul Islam, S M Mahedy Hasan, Md Rakib Hossain, Md Palash Uddin and Md Al Mamun.

To: PLOS ONE Editor-in-Chief

Re: Responses to reviewers

Dear Editor-in-Chief,

We sincerely thank the academic editor and the reviewer for their valuable time and insightful feedback on our previously revised manuscript. We have carefully considered all the comments provided, recognizing that the previous version did not fully meet expectations. We have made the necessary revisions to address these concerns. This document outlines how we have responded to the editor's and reviewer's suggestions. For the ease of the editor and reviewer, all changes in the revised manuscript are highlighted in red.

Thanks for your consideration!

Kind Regards,

Dr. Md Palash Uddin (On behalf of all co-authors)

To the Academic Editor

Comment #1: Please review your reference list to ensure that it is complete and correct. If you have cited papers that have been retracted, please include the rationale for doing so in the manuscript text, or remove these references and replace them with relevant current references. Any changes to the reference list should be mentioned in the rebuttal letter that accompanies your revised manuscript. If you need to cite a retracted article, indicate the article's retracted status in the References list and also include a citation and full reference for the retraction notice.

Author response: Thank you for your valuable reminder regarding the accuracy and integrity of the reference list.

Author action: We have thoroughly reviewed all the references cited in our manuscript to ensure completeness and correctness in accordance with the journal's guidelines. During this process, we identified that none of the cited references have been retracted. However, several necessary corrections and updates have been made to the reference list to improve accuracy and ensure contextual relevance. Specifically, Reference 2 in the initial manuscript was incorrectly cited and has now been corrected. Additionally, Reference 4 was previously cited in an inappropriate context and has been relocated and cited correctly as Reference 7 in the revised manuscript. References 29, 30, and 31, which relate to dataset citations, were previously incomplete and have now been properly corrected while retaining their original numbering. Furthermore, we have added new References 32 to 36 to support the inclusion of three additional datasets introduced in the revised manuscript for extended evaluation. As a result, the original References 32, 33, and 34 have been renumbered as 37, 38, and 39, respectively. Lastly, to support the newly incorporated Grad-CAM-based evaluation, we have included an additional citation, which now appears as Reference 40. All these changes have been carefully reflected in the revised manuscript and are also documented in this rebuttal letter for clarity and transparency.

Additional Comments:

Comment#2: The model primarily depends on publicly sourced data, which may introduce biases and inconsistencies in data quality. This limits its applicability to diverse global waste management scenarios, as the datasets may not fully capture variations in waste profiles across different regions.

Author response: We sincerely thank the reviewer for their thoughtful observation.

Author action: We fully acknowledge that our model is primarily depends on publicly sourced datasets, which inherently introduces risks related to bias and inconsistencies. A significant concern in this context is class imbalance, where certain categories have a disproportionate number of samples compared to others. This imbalance can lead to the model being biased toward the more prevalent classes. This was particularly evident in the "TrashNet" dataset, where the "Trash" class was significantly underrepresented. As a result, the model performed well across most classes but struggled with the "Trash" category. To address this, we applied targeted data augmentation specifically to the "Trash" class to increase its sample size and create a more balanced class distribution. This adjustment led to a notable improvement in model performance across all classes, reducing bias toward the majority classes. A detailed comparative analysis of class-wise performance before and after augmentation is provided in the revised manuscript under the section "Performance Analysis of TrashNet Dataset" (pages 19–20, lines 733–754). While this approach introduces additional complexity, it provides a viable solution for similar challenges in other datasets, should they arise.

Regarding image quality inconsistencies, which arise from the varied sources of images, such as web scraping and real-world devices (e.g., mobile cameras), we made a conscious decision to train the model using raw, unprocessed images from all seven datasets. Apart from resizing, no additional preprocessing or enhancement was applied. In the initial manuscript, this approach is mentioned in the first paragraph of the "Research Methodology" section on page 6. In the revised manuscript, we have further clarified this decision

in the first paragraph of the same section on page 7, emphasizing our intent to evaluate the model's performance under realistic conditions using raw, unmodified images. Our rationale behind this choice was that if the model performs well with raw, unenhanced images, it would likely perform even more robustly with enhanced or cleaner datasets. By using raw images, we aim to better prepare the model for real-world deployment, where image quality can be variable. Additionally, we took great care in thoroughly inspecting the datasets for labeling consistency and can confidently confirm that no labeling errors were present.

To enhance the model's applicability across diverse waste profiles, we expanded our study by incorporating three additional datasets, thereby increasing the total number of unique waste classes to 27. These additions, detailed on page 6-7 (lines 267–289) of the revised manuscript, significantly broaden the scope and diversity of waste categories evaluated. The performance on these new datasets was also encouraging, as reported in the "Validation Across Additional Datasets" subsection (page 21), along with a performance metrics table showcasing the model's effectiveness across these diverse waste classes. As mentioned in the manuscript, most previous studies rely on a single dataset; therefore, our work addresses this limitation by incorporating more diverse datasets to better showcase the model's potential, which has been our goal from the beginning. While it is practically infeasible to represent every region-specific waste variation, our aim was to develop a robust foundational model trained on widely encountered global waste types. We believe that if the model demonstrates strong performance on such globally representative categories, it can be effectively fine-tuned for region-specific waste profiles when provided with relevant local data. Finally, we recognize that these challenges are not yet entirely resolved. Accordingly, we have outlined future directions in the "Threats to Validity" section (page 30, lines 1124–1132), proposing strategies to further improve model's overall robustness for real-world deployment.

Comment#3: The integration of DenseNet201 with squeeze-and-excitation (SE) mechanisms and parallel CNN branches significantly increases computational complexity. This makes it difficult to deploy in resource-constrained environments, such as mobile or embedded systems.

Author response: Thanks for the comment.

Author action: We acknowledge this issue as well. In the original manuscript, we discussed in the "Threats to Validity" subsection (page 22, lines 844–846) that the extended training period of models may pose challenges in resource-constrained settings, as it indicates that the model requires significant computational resources due to its complexity. In the revised manuscript, we have included the total number of parameters for the proposed model, along with additional deep CNN models that we evaluated, to assist in identifying optimal choices for deployment. This information can be found in Table 11, located in the "Performance Comparison with Different Deep CNN Architectures and State of the Art Researches" subsection of the "Results Analysis" section on page 22 of the revised manuscript. From this table, it is evident that models with significantly fewer parameters do not perform as well as the proposed model. However, we have identified three alternative deep CNN models, which have approximately 1/3 to 1/2 the parameters of the proposed model. While these alternatives do not achieve the same level of accuracy as the proposed model, they still demonstrate good performance across all four datasets. These models can be suitable choices for resource-constrained environments, albeit with a trade-off in accuracy. This discussion can be found in the same subsection on page 22, lines 833–848 of the revised manuscript.

To further assess the feasibility of deploying the model in resource-limited environments, we have included an extended investigation in the newly added subsection titled *"Brief Benchmarking of Model Complexity and Deployment Viability"* (pages 26–27 of the revised manuscript). This section presents a comparative benchmarking of five deep learning models that achieved the highest average accuracy scores, as detailed in Table 11. All models were evaluated under uniform experimental conditions using multiple practical deployment metrics such as inference speed, energy consumption, training duration, and model size. This comparative analysis was conducted to provide a clearer understanding of which models could be viable candidates for deployment in environments with limited computational resources. While the proposed model demonstrates superior performance, its complexity and high computational requirements make it more

appropriate for advanced and high-reliability waste management systems. In contrast, we identified and recommended lighter alternatives that, although slightly less accurate, perform reasonably well and are more suitable for constrained settings. We have also addressed this limitation of our proposed model explicitly in the "Threats to Validity" section (page 30, lines 1140–1145 of the revised manuscript), emphasizing the importance of balancing model accuracy with deployability in real-world applications which we aim to do future.

Comment#4: The study does not thoroughly examine the model's robustness against adversarial attacks or noisy data. This could impact reliability in real-world scenarios where images may have varying quality due to environmental factors

Author response: Thank you for pointing this out.

Author action: We understand the importance of evaluating the model's robustness to adversarial attacks and noisy data, which we had not thoroughly investigated previously. While our initial evaluation used raw datasets in their original form, we recognize that a more rigorous assessment is essential to gauge the model's performance in challenging conditions. To address this, we have introduced a new subsection titled "Robustness Evaluation under Adversarial Attacks and Environmental Variability" in the "Results Analysis" section of the revised manuscript. This subsection evaluates the model's performance under moderate levels of noise, such as salt-and-pepper noise, Gaussian noise, and FGSM adversarial attacks, to simulate real-world scenarios. The results, including relevant performance metrics and accuracy reductions in the presence of noise and adversarial inputs, are presented in Table 9 on page 21, with further discussion in the accompanying subsection. We hope this addresses the concern regarding the model's robustness to adversarial attacks and noisy data.

Comment#5: The system has only been deployed on a local server, and its scalability to larger, more complex systems or across different geographies remains untested. Additionally, real-time classification latency and energy consumption in large-scale deployments have not been evaluated.

Author response: Thank you for highlighting this concern.

Author action: We acknowledge this limitation of our study that the model has only been deployed on a local server, and its scalability to larger, more complex systems or across different geographies remains untested, which we have already addressed in the original manuscript in the "Threats to Validity" subsection (page 22, lines 852–856). In the revised version, this information is now located in the "Threats to Validity" section on page 30 (lines 1148–1153). Moving forward, we aim to explore the potential for deploying our model on edge devices or larger complex systems as currently we don't have that type of resource to do so, as mentioned in the "Conclusion and Future Research" section as well.

In the previous version of the revised manuscript, we introduced a "Brief Overview of Empirical Time Complexity" subsection within the "Results Analysis" section, where we provided a detailed analysis of the model's time complexity across four datasets. This subsection includes the model's inference time, which measures the duration required to make predictions on new data. The analysis, along with a comprehensive description, is available on page 25 in the latest revised manuscript version, offering an estimate of potential latency based on the model's performance on the test set. To better assess real-world latency, we also conducted an end-to-end latency evaluation by making predictions via the website in real time. This analysis includes factors such as image upload time and server processing time, offering a more accurate measure of real-time delay. As expected, the results indicate that end-to-end latency exceeds the model's standalone inference time. This evaluation, with further details, is now included in the "Website Deployment" subsection under the "Real-Time Deployment of Waste Classification System" section on page 29, lines

1090–1100 of the latest revised manuscript. This addition offers a practical perspective on potential latency issues in real-time waste classification. Obviously, we understand that it is not enough to exactly evaluate the result while large-scale deployments, as mentioned we currently don't have such resource to do so, but it definitely will provide a rough idea about how the model may perform.

Regarding energy consumption in large-scale deployments, we evaluated the model's energy consumption and estimated carbon emissions across the five datasets. The findings are summarized in Table 16 on page 26 of the revised manuscript, with further details provided in the newly added "Energy Consumption Assessment" subsection within the "Results Analysis" section. This assessment serves as a preliminary indication of the model's energy efficiency and environmental impact, which are crucial for sustainable applications. In the "Threats to Validity" section and the "Conclusion and Future Research" section, we have noted our intention to focus on broader deployment in future work, which will enable a more comprehensive assessment of the model's energy consumption at scale. Our evaluation of energy consumption, included in the revised manuscript, offers a valuable foundation for understanding the model's energy requirements within our current scope and their implications for environmentally responsible waste management systems. We hope this adequately addresses the editor's concern.

Comment#6: The work does not incorporate visualization techniques for explainability, making it difficult to interpret the model's decision-making process. The lack of explainability tools could hinder trust and adoption by stakeholders who require transparency in AI-based waste classification.

Author response: Thank you for highlighting this concern as well.

Author action: We fully acknowledge the limitation that the original version of our study did not include visualization techniques for model explainability, which can indeed hinder interpretability and reduce stakeholder trust in AI-based waste classification systems. Recognizing the importance of transparency, we have now incorporated Grad-CAM (Gradient-weighted Class Activation Mapping) visualizations into our study. This enhancement offers a clearer understanding of how the model arrives at its predictions on unseen data, thereby reinforcing the interpretability and reliability of the system. To address this, we have added a dedicated subsection titled "Model Explainability via Grad-CAM" within the "Results Analysis" section of the revised manuscript. This section provides deeper insights into the internal decision-making process of the proposed DenseNet201-infused parallel SE-CNN model. We believe this addition significantly enhances the transparency of the model's behavior and helps build trust among potential users and stakeholders. We hope this adequately mitigates the editor's concern regarding the lack of explainability tools.

To the Reviewer:

Comment#1: Areas for Improvement:
Dataset representation: While four datasets are used, there could be deeper discussion on how well these represent global waste variations, especially in regions with unique waste profiles. Consider addressing whether certain geographic-specific waste types might be underrepresented.

Author response: We appreciate the feedback on this important aspect.

Author action: We appreciate the reviewer's valuable observation. While our original work utilized four datasets to evaluate the model, we have now extended the study by incorporating three additional publicly available datasets. This expansion increases the diversity of waste types analyzed, bringing the total number of unique waste classes to 27. These updates are discussed in detail on pages 6–7 (lines 267–289) of the revised manuscript. To ensure a broad and realistic scope, we also cited relevant studies that support the notion that these selected waste categories encompass the most commonly encountered global waste types. This approach allows the model to reflect a wide range of real-world waste scenarios.

Performance evaluations on the newly added datasets are presented in the revised manuscript under the "Validation Across Additional Datasets" subsection (page 21), along with a comprehensive table of performance metrics that confirm the model's consistency across different data domains. In contrast to most prior studies that focus on a single dataset, our intention has been to overcome this limitation by demonstrating the model's generalizability through a broader evaluation strategy. Although we acknowledge that it is not feasible to account for every geographically unique waste stream, our aim has been to establish a generalized and reliable baseline model trained on globally relevant categories. This foundation could then be fine-tuned using localized datasets, allowing adaptation to specific regional waste profiles.

We recognize that the challenge of global generalization is ongoing and cannot be entirely solved within a single study. Therefore, we have highlighted this issue in the "Threats to Validity" section (page 30, lines 1124–1132), and suggested future efforts to further enhance the model's robustness, adaptability, and readiness for real-world deployment in a wide variety of settings.

Comment#2: Image quality considerations: The performance on varying image qualities is not extensively discussed. Since real-world applications would encounter images of varying qualities, this deserves more attention.

Author response: We appreciate the feedback on this important aspect.

Author action: We sincerely appreciate your insightful feedback regarding image quality, a factor that plays a critical role in real-world deployment. As noted in our earlier version, our model was originally trained and tested using raw, unfiltered images to reflect natural, uncurated data environments. However, we fully agree that a more deliberate and systematic evaluation of the model's resilience to image quality variations is essential to truly assess its robustness under practical conditions. To address this concern, we extended our experiments by simulating a range of real-world image quality degradations. These include controlled modifications in brightness, contrast, saturation, hue, and gamma correction, along with subtle random sharpening and blurring. These transformations were applied within a realistic range to mimic the types of variability typically encountered due to lighting, environmental interference, and device differences in practical field applications. The impact of these variations on model performance is thoroughly analyzed in the newly added subsection, "Robustness Evaluation under Adversarial Attacks and Environmental Variability", within the "Results Analysis" section (pages 20–21 of the revised manuscript).

Furthermore, the outcomes of this analysis are quantitatively presented in Table 9 (page 21), where we report changes in accuracy due to each specific variation. This addition provides a more comprehensive understanding of the model's behavior under suboptimal imaging conditions. We hope this extended evaluation convincingly addresses your concerns and illustrates our commitment to ensuring that the model is adaptable and resilient in real-world deployment scenarios.

Comment#3: Computational efficiency: While the empirical time complexity is briefly addressed, a more detailed analysis of computational requirements would strengthen the paper, especially considering deployment scenarios with limited computational resources.

Author response: We sincerely thank the reviewer for this valuable suggestion.

Author action: We are grateful to the reviewer for emphasizing the importance of a detailed computational efficiency analysis—an essential aspect when considering real-world deployments, particularly in environments with limited computational capacity. Although our previous version of the manuscript briefly addressed empirical time complexity, we acknowledged the need for a more thorough evaluation to better reflect practical deployment scenarios. Accordingly, we have expanded our study by introducing a dedicated subsection titled *"Brief Benchmarking of Model Complexity and Deployment Viability"* (pages 26–27 of the revised manuscript). This section presents a comprehensive benchmarking of the five top-performing models, selected based on their average classification accuracy (as reported in Table 11). Each model was evaluated under uniform experimental conditions across several deployment-relevant metrics, including inference latency, training duration, energy usage, and model size. The comparative results are detailed in Table 17. Our findings indicate that the proposed DenseNet201-infused SE-CNN model, while achieving the highest predictive accuracy, entails greater computational demands, making it more suitable for robust, high-end waste management infrastructures. In contrast, we also identified more lightweight alternatives that, despite a modest reduction in accuracy, offer significantly better efficiency and are more appropriate for constrained environments. We have also addressed this limitation of our proposed model explicitly in the "Threats to Validity" section (page 30, lines 1140–1145 of the revised manuscript), emphasizing the importance of balancing model accuracy with deployability in real-world applications which we aim to do future.

Comment#4: Model interpretability: The proposed architecture is complex, which might make interpretation of its decisions challenging. Some discussion on explainability methods that could complement this system would be valuable.

Author response: We thank the reviewer for this valuable suggestion.

Author action: We fully recognize that the complexity of the proposed architecture can pose challenges in interpreting its decision-making process. In response, and to enhance the transparency of our model, we have integrated Gradient-weighted Class Activation Mapping (Grad-CAM) into our study. This interpretability tool enables us to visualize the regions of the input image that most strongly influence the model's predictions, offering a more intuitive understanding of its behavior on previously unseen data. To reflect this improvement, we have included a new subsection titled "Model Explainability via Grad-CAM" within the Results Analysis section of the revised manuscript. This section provides a detailed examination of how the proposed DenseNet201-infused parallel SE-CNN model processes and prioritizes visual information during classification. We hope this addition effectively addresses the reviewer's concern regarding the need for explainability tools.

Comment#5: Long-term performance stability: The paper doesn't address how the model might perform over time as waste compositions change. A brief discussion on potential strategies for model updating would enhance the practical relevance.

Author response: Thank you for highlighting this issue.

Author action: We fully acknowledge the reviewer's insightful observation regarding the long-term performance stability of the model as waste compositions evolve over time. This is indeed a critical concern, especially with the frequent introduction of new packaging formats, material substitutions, and recycling practices, which can significantly alter the visual and structural characteristics of waste. While our model has been evaluated for robustness under conditions such as adversarial perturbations, noisy data, and various environmental distortions—allowing it to perform reliably even with degraded image quality—these measures alone are insufficient to address shifts in the fundamental composition of waste. When such changes occur, the model's predictive reliability may degrade, as it may encounter classes or features it was never trained to recognize. To tackle this issue, we recognize the necessity of adopting continual learning strategies, which would allow the model to adapt over time without requiring complete retraining. In the revised manuscript, we have included a brief discussion of potential strategies to address this challenge in the "Threats to Validity" section (page 30, lines 1133–1139). We believe this addition highlights a forward-looking perspective and enhances the practical relevance of the work.

Comment#6: Technical Suggestions:
The confusion matrices in Figures 5, 9, 13, and 17 provide valuable insights, but further analysis of the specific misclassifications (especially between similar materials like paper and cardboard) would strengthen the discussion.

Author response: Thank you for highlighting this important aspect.

Author action: We appreciate the reviewer's insightful suggestion regarding a deeper analysis of specific misclassifications, particularly between visually similar classes such as paper and cardboard. Upon a detailed examination of the results across our four primary datasets, it became evident that the TrashNet dataset exhibited a notably higher rate of misclassification—especially in the Cardboard and Trash classes. As reflected in Table 7 of the revised manuscript, this issue stemmed from two primary factors: (i) visual similarity between cardboard, paper, and certain trash items, and (ii) a significant class imbalance, particularly for the underrepresented Trash category. Consequently, while the model maintained high accuracy across most classes, its performance degraded when attempting to distinguish between these closely related categories. To address this issue, we implemented targeted data augmentation strategies aimed at balancing the class distribution, particularly enhancing representation for the underperforming classes. This not only mitigated the imbalance but also enriched the model's ability to generalize patterns between visually similar materials. The improved class-wise performance—post augmentation—is documented in Table 8 and discussed in detail under the section "Performance Analysis of TrashNet Dataset" (pages 19–20, lines 733–754) of the revised manuscript. While this augmentation introduces added complexity to the preprocessing pipeline, it offers a practical and effective approach for reducing misclassification—especially among classes with subtle visual differences. We believe this enhancement meaningfully addresses the reviewer's concern.

Comment#7: Consider including a brief ablation study to quantify the specific contributions of the parallel CNN branches and the SE attention mechanism to the overall performance improvement.

Author response: Thank you for raising this important issue.

Author action: We fully acknowledge the importance of evaluating the individual contributions of the architectural components—specifically, the parallel CNN branches and the SE attention mechanism—to better understand their roles in driving the performance gains of the proposed model. To this end, we have introduced an ablation study presented in Table 14, titled *"Impact of Parallel CNN Branches and SE Attention on Model Performance."* In this study, we evaluated four key model variants in addition to the full proposed architecture. These variants isolate the presence or absence of the parallel CNN branches and SE attention mechanism, allowing us to quantify their individual and combined effects. The results clearly demonstrate the additive and synergistic benefits of both components, with the full model yielding the highest performance across metrics. This detailed analysis and its discussion can be found in the revised manuscript on pages 24–25, lines 900–930. We believe this ablation study provides valuable insights into the architecture's design rationale and hope it sufficiently addresses the reviewer's concern.

Comment#8: The empirical time complexity analysis in Table 10 is useful, but could be expanded to include comparisons with other architectures to provide context for deployment considerations.

Author response: Thank you for pointing this out.

Author action: We fully recognize that, beyond the empirical time complexity assessment provided in Table 10, comparing our model with other architectures is essential to offer a clearer perspective on deployment feasibility. To address this, and as previously mentioned, we have introduced a new subsection titled "Brief Benchmarking of Model Complexity and Deployment Viability" (pages 26–27 in the revised manuscript). This section presents an in-depth comparative analysis of the five highest-performing models, selected based on their average classification accuracy as shown in Table 11. Each model was tested under consistent experimental conditions and evaluated using several deployment-critical metrics, such as inference time, training duration, energy efficiency, and model footprint. The results of this benchmarking effort are presented in Table 17. We believe that such a multi-faceted analysis goes beyond time complexity alone and offers a more practical and holistic view of the trade-offs between accuracy and deployability—particularly for real-world, resource-constrained scenarios. We hope this addresses the reviewer's concern and adds valuable context to our study.

Comment#9: The web implementation described in Section 6.1 could benefit from more details on potential latency issues in real-time classification scenarios.

Author response: Thank you for highlighting this concern.

Author action: We appreciate the reviewer's suggestion and have taken steps to address the concern regarding latency in real-time classification scenarios. To provide a more grounded perspective on potential delays, we performed an end-to-end latency assessment using our deployed website. This analysis considered both image upload durations and backend server processing times, offering a more realistic picture of the total response time users may experience. As anticipated, the total end-to-end latency is naturally higher than the standalone inference time of the model. The results and discussion of this evaluation have been incorporated into the "Website Deployment" subsection, within the broader section titled *"Real-Time Deployment of Waste Classification System"* (page 29, lines 1090–1100 of the revised manuscript). While we acknowledge that a complete simulation of latency under large-scale deployment conditions would require additional infrastructure, which is currently beyond our capacity, this evaluation nonetheless provides an informative approximation of real-world performance. We hope this added analysis offers helpful insight into practical latency considerations and adequately addresses the reviewer's concern.

Comment#10: Section 6.3 on threats to validity provides good insights, but could be expanded to address potential issues with model generalization to unseen waste types or novel categories.

Author response: Thanks for the suggestion.

Author action: We acknowledge the critical importance of ensuring that the model can generalize effectively to previously unseen waste types or novel categories—especially given the dynamic nature of waste streams. In response to this concern, we have expanded the "Threats to Validity" section to address this limitation more explicitly. We note that when the model encounters unfamiliar waste items not represented in the training data, it may result in misclassification or low prediction confidence. To mitigate this challenge, we have proposed several forward-looking strategies aimed at improving the model's adaptability. These considerations are now briefly added in the revised manuscript on page 30, lines 1133–1139. We believe this addition adds depth to the discussion and outlines a practical path forward for enhancing model generalization in real-world applications.
